
JOURNAL INFORMATION
==============================
NLM Title Abbreviation: Sci China Life Sci
Journal ID: Sci China Life Sci
NLM Journal ID: 101529880

Science China. Life Sciences

ISSN: 1674-7305
EISSN: 1869-1889

ARTICLE INFORMATION
==============================
PMCID: PMC7089048
PMID: 23238747
DOI: 10.1007/s11427-012-4408-6
Article ID: 4408
Article version: 1
Subjects: News and Views

‘One Health’ for the people of Hong Kong and the world

Kok Kin-Hang
Tang Hei-Man Vincent
Jin Dong-Yan dyjin@hku.hk

grid.194645.b 0000000121742757 Department of Biochemistry, University of Hong Kong, Hong Kong, China
Electronic publication date: 2012 Dec 12
Print publication date: 2016
Volume: 59
Issue: 10
First page: 1068
Last page: 1070
Copyright: © The Author(s) 2016
License: Open AccessThis article is distributed under the terms of the Creative Commons Attribution 2.0 International License (https://creativecommons.org/licenses/by/2.0), which permits unrestricted use, distribution, and reproduction in any medium, provided the original work is properly cited.
License URL: https://creativecommons.org/licenses/by/2.0/

Keywords: Imatinib, Severe Acute Respiratory Syndrome, Dasatinib, Nilotinib, Severe Acute Respiratory Syndrome
issue-copyright-statement: © Science in China Press and Springer-Verlag GmbH 2016

==============================

Kin-Hang Kok, Hei-Man Vincent Tang & Dong-Yan Jin*

Translational medicine refers to the conversion of scientific discoveries into improvements in medical practice. ‘From bench to bedside and back’ is a phrase that is often used to describe the two-way information flow in translational medicine. On one hand, scientists working at the bench make new discoveries that enable clinicians to revolutionize patient care, while on the other hand, clinical investigations at the bedside provide new knowledge about diseases, which stimulate basic research at the bench.

One recent success story that illustrates the ‘from bench to bedside and back’ nature of translational medicine concerns the development of small-molecule inhibitors of the Bcr-Abl fusion oncoprotein as therapeutics for chronic myeloid leukemia (CML). The discovery of the Philadelphia chromosome by Janet Rowley in 1973 and the study of the Bcr-Abl fusion oncoprotein expressed exclusively in CML cells finally led to the rational design and development of imatinib for clinical use. Imatinib is an ATP analog that impedes the binding of ATP to Bcr-Abl tyrosine kinase and thereby inhibits its oncogenic activity. Initial therapy of CML with imatinib results in complete remission and minimal side effects in approximately 70% of patients. The success of imatinib is rare in the history of cancer medicine and represents the start of a new era of molecularly-targeted cancer therapy. However, the potency of imatinib to inhibit Bcr-Abl is relatively low and resistance develops in patients through various new mutations in Bcr-Abl. These clinical investigations at the bedside stimulated new directions for basic research at the bench. The consequent biochemical, molecular and pharmacological studies to address these questions have given rise to the second-generation Bcr-Abl inhibitors dasatinib and nilotinib, which are superior to imatinib. These inhibitors are 20- to 300-fold more potent than imatinib in inhibiting Bcr-Abl. In particular, the conformation-dependent binding of dasatinib to Bcr-Abl renders the development of resistance through single point mutations more difficult. Dasatinib and nilotinib are therefore recommended for clinical use at the bedside as front-line therapy for CML (Druker 2008; Hantschel et al., 2012).

Translational medicine aspires to bridge the gap between basic and clinical medicine, which involves the daunting task of breaking down existing barriers between biomedical scientists and clinicians. These barriers are particularly strong in mainland China, where biomedical science and clinical medicine have developed into separate professions. Communication among scientists and clinicians from different disciplines and institutions has to be re-established and substantially enhanced with the aim of cross-fertilizing new ideas, strategies and innovations in disease prevention and intervention. In keeping with this main theme of translational medicine, we would like to go one step further by introducing and promoting the concept of ‘One Health’ (Osburn et al., 2009).

The goals of the ‘One World-One Medicine-One Health’ initiative are to “promote, improve, and defend the health and well-being of all species by enhancing cooperation and collaboration between physicians, veterinarians, other scientific health and environmental professionals” (http://www.onehealthinitiative.com/). The idea is to expand the dialogues and collaborations in “all aspects of health care for humans, animals and the environment”. These collaborations must be coequal and all inclusive. They should engage physicians, osteopaths, veterinarians, dentists, nurses as well as other scientific, health and environmentally-related disciplines. In reality, uniting human and veterinary medicine represents another daunting challenge in mainland China for historical and other reasons. In one extreme case, human virologists were prohibited by law from touching dead birds infected by the H5N1 influenza virus found in the area near Qinghai Lake.

One fundamental issue in translational medicine and in the 'One Health' initiative is the integration of basic and clinical medicines. Hong Kong, as the gateway from China to the world and vice versa, is not only a city where East meets West, but also a place where modern medicine was introduced to save the lives of many people. Looking back in history, several major medical discoveries that were initially made in Hong Kong in the past decades have had significant and long-lasting impacts on the world. Notably, pioneering clinician scientists in Hong Kong have already set excellent examples for the integration of basic and clinical medicines. In this perspective, they were also exemplary practitioners of translational medicine or 'One Medicine'. What we need to do in the pursuit of translational medicine or 'One Health' in today's changing world, is to learn from these pioneers in Hong Kong's history. Here we review the anecdotal records of Sir Patrick Manson's pioneering work on Filaria in the 1880s and the discovery of severe acute respiratory syndrome (SARS) coronavirus in 2003.

Sir Patrick Manson (1844–1922) was the founding Dean of the Hong Kong College of Medicine for Chinese in 1886, which became the Faculty of Medicine of the University of Hong Kong in 1911. Dr. Sun Yat-sen was one of Manson's first medical students and Manson also used his influence in 1896 to ensure the release of Sun from custody by Qing officials in London. Manson practiced medicine privately in Hong Kong in 1883–1889. Before he came to Hong Kong, he also saw patients in Taiwan for 5 years and in Xiamen for 13 years. Manson was a great physician and surgeon who was already famous in Taiwan for performing surgery on a 19-year-old man suffering from elephantiasis caused by Filaria. He saw patients on a daily basis and saved the lives of many people infected with parasites using his medical and surgical techniques. Fascinated by these medically important research questions that he encountered during his private practice in Hong Kong, Manson later went back to London to found the London School of Tropical Medicine, where he carried out parasitological research that led to the identification of *Schistosoma mansoni*, the cause of a common form of schistosomiasis. He was also instrumental in providing the first evidence for the involvement of an arthropod vector in the life cycle of a parasite. Under his supervision, Ronald Ross fully documented the role of mosquitoes as a vector of malaria and was awarded the Nobel Prize in 1902 for this discovery. All these research findings are truly ground-breaking and have had a tremendous impact on patient care and the practice of medicine, as well as disease prevention and control. Manson was therefore known as the 'Father of Tropical Medicine' (Jay 2000; Lai et al., 2003).

In the past 120 years, the tradition of pursuing excellence in patient care and medical research, which can be traced back to the early life and work of Sir Patrick Manson, has been carried on by generations of scientists and clinicians in Hong Kong. This was well illustrated when Hong Kong was hit by SARS in 2003. It is now unequivocally accepted that the SARS coronavirus, which is responsible for SARS, was discovered by a group of scientists and clinicians at the University of Hong Kong led by Prof. Malik Peiris and Prof. K.Y. Yuen (Peiris et al., 2003). In our opinion, this was accomplished at this university rather than elsewhere for several reasons. First, the leading virologists and clinicians in this group had the expertise and management skills for tackling medically important problems using a multidisciplinary approach. Second, the University of Hong Kong has a strong infrastructure that facilitates the discovery of new etiologic agents, including a well-integrated research unit in which clinical samples are collected and various kinds of microbiological assays are systemically performed. This organization is perfectly suited to the purpose of clinical and translational microbiological research. Third, the group had learned from previous success in the study of influenza viruses. It is particularly noteworthy that the H5N1 influenza virus was also identified by the same group (Yuen et al., 1998). Finally, the group was small but functional, and familiar not only with the most advanced molecular and high-throughput technologies, but also able to employ all the classical methods for virus isolation and characterization. Of note, the success in identifying the SARS coronavirus was attributed to neither 'big science' nor 'big grants'. They succeeded in the face of fierce competition from American and European groups only because they were good and because they used the right approach. Ten years after the identification of the SARS coronavirus, many other groups that jumped into the field after the outbreak of SARS have left, while the teams at the University of Hong Kong continued to make major contributions to the field. The recently identified group 2c human coronavirus EMC associated with one severe and two fatal cases of respiratory syndrome in Saudi Arabia was most closely related to two bat coronaviruses identified by Hong Kong scientists in 2007 (Woo et al., 2007; Zaki et al., 2012). Thus, the findings obtained by scientists at the University of Hong Kong have shaped the field of coronavirus research for years to come. Their ground-breaking and continuing work in the field has been translated into new strategies, reagents and assays for the prevention and control of infectious diseases. In this sense, they have provided another successful example of the 'from bench to bedside and back' model in translational medicine and 'One Health'.

The integration of basic and clinical medicine is highly characteristic of the life and work of Sir Patrick Manson who founded the Medical Faculty of the University of Hong

Kong and the London School of Tropical Medicine. It was also the key to the success in the identification of the SARS coronavirus in Hong Kong about 110 years later. This should also be the guiding principle of translational medicine and 'One Health' as well. The ultimate goal of both is to promote human health and well being. To this end, free access to information, samples and resources is of fundamental importance. If Manson had not tested his great ideas that originated from his medical practices in Hong Kong, Taiwan and Xiamen with carefully designed experiments in mosquitoes, he would never have made the discoveries that later transformed modern medicine. If Prof. Yuen and colleagues in the University of Hong Kong had not had access to the wild animals with the help of veterinarians and other professionals, they would never have traced the SARS-like coronaviruses to bats (Lau et al., 2005). Learning from the successful experience of pioneering scientists who worked in Hong Kong will help identify major deficiencies in the infrastructure for medical research in mainland China. Cracking the institutional and disciplinary barriers that hamper communication and collaboration between scientists and clinicians is an essential prerequisite for the future development of translational medicine and 'One Health'.

**Compliance and ethics** *The author(s) declare that they have no conflict of interest.*

**Open Access** This article is distributed under the terms of the Creative Commons Attribution License which permits any use, distribution, and reproduction in any medium, provided the original author(s) and source are credited.

This article is published with open access at link.springer.com


References

Druker B.J. Translation of the Philadelphia chromosome into therapy for CML. Blood 2008 112 4808 4817 10.1182/blood-2008-07-077958 19064740
Hantschel O. Grebien F. Superti-Furga G. The growing arsenal of ATP-competitive and allosteric inhibitors of BCR-ABL. Cancer Res 2012 72 4890 4895 10.1158/0008-5472.CAN-12-1276 23002203 PMC3517953
Jay V. Sir Patrick Manson: Father of tropical medicine. Arch Pathol Lab Med 2000 124 1594 1595 11079007 10.5858/2000-124-1594-SPM
Lai K.K.Y. Chan J.L.Y. Lai K.K.T. Schmer G. Fritsche T.R. Sir Patrick Manson: good medicine for the people of Hong Kong. Hong Kong Med J 2003 9 145 147 12668830
Lau S.K. Woo P.C. Li K.S. Huang Y. Tsoi H.W. Wong B.H. Wong S.S. Leung S.Y. Chan K.H. Yuen K.Y. Severe acute respiratory syndrome coronavirus-like virus in Chinese horseshoe bats. Proc Natl Acad Sci USA 2005 102 14040 14045 10.1073/pnas.0506735102 16169905 PMC1236580
Osburn B. Scott C. Gibbs P. One world-one medicine-one health: emerging veterinary challenges and opportunities. Rev Sci Tech 2009 28 481 486 20128454 10.20506/rst.28.2.1884
Peiris J.S. Lai S.T. Poon L.L. Guan Y. Yam L.Y. Lim W. Nicholls J. Yee W.K. Yan W.W. Cheung M.T. Cheng V.C. Chan K.H. Tsang D.N. Yung R.W. Ng T.K. Yuen K.Y. SARS study group Coronavirus as a possible cause of severe acute respiratory syndrome. Lancet 2003 361 1319 1325 10.1016/S0140-6736(03)13077-2 12711465 PMC7112372
Woo P.C. Wang M. Lau S.K. Xu H. Poon R.W. Guo R. Wong B.H. Gao K. Tsoi H.W. Huang Y. Li K.S. Lam C.S. Chan K.H. Zheng B.J. Yuen K.Y. Comparative analysis of twelve genomes of three novel group 2c and group 2d coronaviruses reveals unique group and subgroup features. J Virol 2007 81 1574 1585 10.1128/JVI.02182-06 17121802 PMC1797546
Yuen K.Y. Chan P.K. Peiris M. Tsang D.N. Que T.L. Shortridge K.F. Cheung P.T. To W.K. Ho E.T. Sung R. Cheng A.F. Clinical features and rapid viral diagnosis of human disease associated with avian influenza A H5N1 virus. Lancet 1998 351 467 471 10.1016/S0140-6736(98)01182-9 9482437
Zaki A.M. van Boheemen S. Bestebroer T.M. Osterhaus A.D. Fouchier R.A. Isolation of a novel coronavirus from a man with pneumonia in Saudi Arabia. N Engl J Med 2012 367 1814 1820 10.1056/NEJMoa1211721 23075143
